# Supplementary material for: Causal Relationship between Aging and Anorexia Nervosa: A White-Matter-Microstructure-Mediated Mendelian Randomization Analysis
Source: Biomedicines. 2024 Aug 16;12(8):1874. doi: 10.3390/biomedicines12081874 (PMC11351342; doi:10.3390/biomedicines12081874)
Supplement: Supplementary file 1 [file biomedicines-12-01874-s001.zip › Supplementary Figure/Figure S4/Singe SNP plot of MR analysis for TL to ubm-b-1997.pdf]

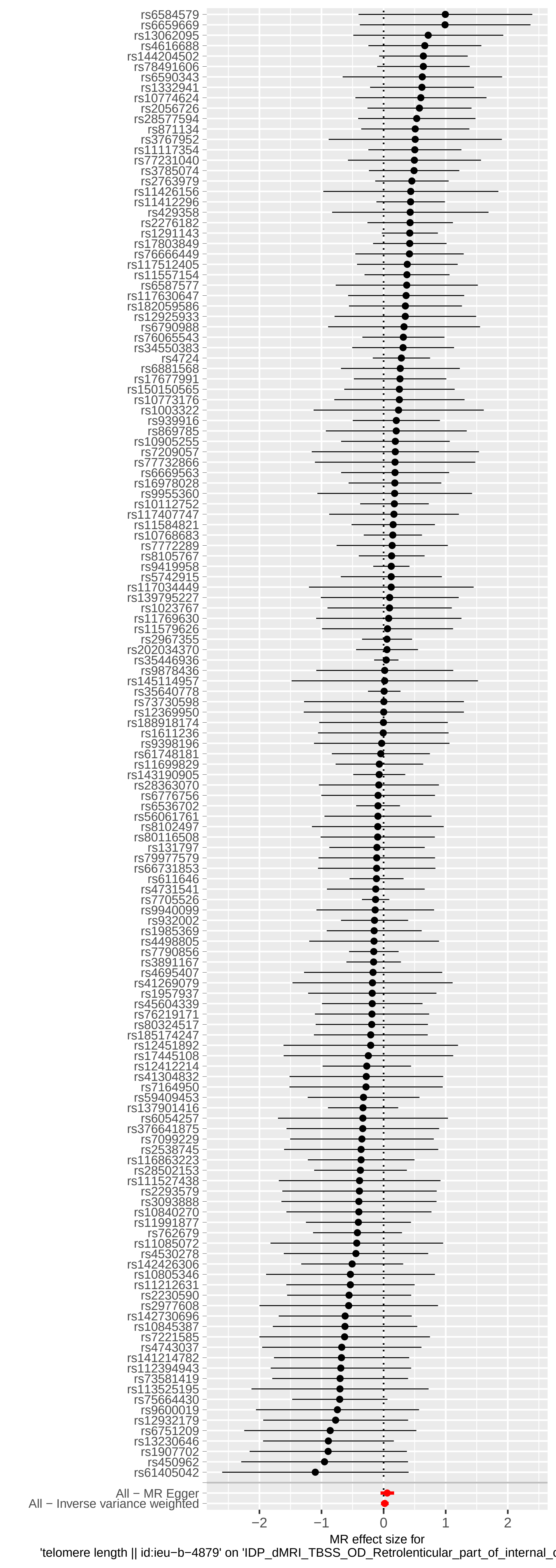
rs6584579
rs6659669
rs13062095
rs4616688
rs144204502
rs78491606
rs6590343
rs1332941
rs10774624
rs2056726
rs28577594
rs871134
rs3767952
rs11117354
rs77231040
rs3785074
rs2763979
rs11426156
rs11412296
rs429358
rs2276182
rs1291143
rs17803849
rs76666449
rs117512405
rs11557154
rs6587577
rs117630647
rs182059586
rs12925933
rs6790988
rs76065543
rs34550383
rs4724
rs6881568
rs17677991
rs150150565
rs10773176
rs1003322
rs939916
rs869785
rs10905255
rs7209057
rs77732866
rs6669563
rs16978028
rs9955360
rs10112752
rs117407747
rs11584821
rs10768683
rs7772289
rs8105767
rs9419958
rs5742915
rs117034449
rs139795227
rs1023767
rs11769630
rs11579626
rs2967355
rs202034370
rs35446936
rs9878436
rs145114957
rs35640778
rs73730598
rs12369950
rs188918174
rs1611236
rs9398196
rs61748181
rs11699829
rs143190905
rs28363070
rs6776756
rs6536702
rs56061761
rs8102497
rs80116508
rs131797
rs79977579
rs66731853
rs611646
rs4731541
rs7705526
rs9940099
rs932002
rs1985369
rs4498805
rs7790856
rs3891167
rs4695407
rs41269079
rs1957937
rs45604339
rs76219171
rs80324517
rs185174247
rs12451892
rs17445108
rs12412214
rs41304832
rs7164950
rs59409453
rs137901416
rs6054257
rs376641875
rs7099229
rs2538745
rs116863223
rs28502153
rs111527438
rs2293579
rs3093888
rs10840270
rs11991877
rs762679
rs11085072
rs4530278
rs142426306
rs10805346
rs11212631
rs2230590
rs2977608
rs142730696
rs10845387
rs7221585
rs4743037
rs141214782
rs112394943
rs73581419
rs113525195
rs75664430
rs9600019
rs12932179
rs6751209
rs13230646
rs1907702
rs450962
rs61405042
All − MR Egger
All − Inverse variance weighted
−2
−1
0
1
2
MR effect size for
'telomere length || id:ieu−b−4879' on 'IDP_dMRI_TBSS_OD_Retrolenticular_part_of_internal_c
